# Hydrogel-driven innovations for targeted delivery, immune modulation, and tissue repair in thyroid cancer therapy

**DOI:** 10.3389/fcell.2025.1608709

**Published:** 2025-07-25

**Authors:** Hanxiao Tang, Yingli Tao, Yongsheng Zhang, Yun Ling, Yunjie Sheng, Lingya Yu

**Affiliations:** ^1^ Department of Pharmacy, Tongde Hospital of Zhejiang Province, Hangzhou, China; ^2^ Department of Reproductive Immunology, Tongde Hospital of Zhejiang Province, Hangzhou, China; ^3^ School of Basic Medical Sciences, Zhejiang Chinese Medical University, Hangzhou, China; ^4^ Animal Experimental Research Center, Zhejiang Chinese Medical University, Hangzhou, China; ^5^ School of Pharmaceutical Sciences, Zhejiang Chinese Medical University, Hangzhou, China; ^6^ Department of Pediatrics, Tongde Hospital of Zhejiang Province, Hangzhou, China

**Keywords:** hydrogel, thyroid cancer, drug delivery system, stimulus response, targeted controlled release, immunotherapy, tissue engineering reconstruction

## Abstract

**Background:**

Thyroid cancer is the fastest-growing endocrine malignancy globally, with an increasing incidence in younger patients. Conventional therapies, including surgery, radioactive-iodine (RAI) ablation, endocrine suppression, and multi-kinase inhibition, have improved outcomes but are limited by peri-operative morbidity, systemic toxicity, long treatment durations, and the development of drug resistance.

**Objective:**

This review synthesizes current advancements in hydrogel-based therapy, focusing on its potential as a multifunctional platform to overcome the challenges in thyroid cancer management. The review highlights the role of hydrogels not only as carriers for various drugs but also as specific agents for thyroid cancer treatment, offering targeted delivery, immune modulation, and tissue repair.

**Content:**

Modern hydrogels, with their high biocompatibility, tunable mechanical properties, and responsiveness to external stimuli (pH, temperature, light, enzymes), provide site-specific, sustained release of chemotherapeutics, tyrosine kinase inhibitors (TKIs), and ^131^I. This localised drug delivery increases tumor exposure while sparing vital cervical structures, a critical advantage in thyroid cancer therapy. Composite and in situ-forming hydrogels can also modify the tumour-immune microenvironment, delivering cytokines, checkpoint inhibitors, or vaccine adjuvants to transform immune “cold” lesions, such as poorly differentiated thyroid carcinoma (PDTC), into “hot” immune-responsive sites. Additionally, 3D hydrogel matrices mimic the extracellular matrix, aiding in post-resection tissue repair, preventing cervical adhesions, and enabling the bioprinting of thyroid organoids and CAR-T systems. When integrated with photothermal and photodynamic agents, hydrogels provide synergistic tumour ablation, while formulations with nanosilver or antibiotics help reduce the risk of post-surgical infection.

**Challenges and Outlook:**

Several challenges remain, including optimising the degradation kinetics of hydrogels without compromising their mechanical integrity, improving the loading of hydrophobic TKIs, and better understanding the interactions between hydrogels, the immune system, and tumour tissues *in vivo*. Large, multi-centre trials are needed to confirm the long-term safety of hydrogel-based therapies and establish their superiority over current standard treatments. Future directions will likely focus on developing “smart” multifunctional hydrogels that can co-encapsulate dual-target inhibitors (e.g., BRAFV600E + MEK), PROTACs, oncolytic viruses, and imaging probes, all informed by single-cell omics-guided patient stratification to enhance therapeutic precision.

**Conclusion:**

By integrating precision drug delivery, immune modulation, and tissue engineering into a single platform, hydrogels are positioned to revolutionize the treatment of thyroid cancer. They offer a promising solution for improving locoregional control, minimizing systemic toxicity, and enhancing the survival and quality of life of patients with both differentiated and undifferentiated thyroid cancers. The versatility of hydrogels as carriers for a broad range of therapeutic agents, as well as their specificity for thyroid cancer treatment, highlights their potential to redefine the future of targeted cancer therapies.

## 1 Introduction

Thyroid cancer, the most prevalent malignancy of the endocrine system, has witnessed a significant rise in global incidence, increasingly affecting younger populations across diverse regions (Kim et al., 2020). Conventional treatments—surgical resection, radioactive iodine (RAI) therapy, endocrine modulation, and targeted pharmacotherapy—have improved outcomes but remain hindered by notable limitations (Chen et al., 2024). These include postoperative complications such as tissue adhesion and functional impairment, off-target toxicity, therapeutic resistance, and substantial financial burdens. Moreover, the molecular heterogeneity of thyroid cancer complicates accurate diagnosis and individualized treatment planning. In light of these challenges, hydrogels have emerged as a promising class of smart biomaterials with transformative potential in oncological applications (Zhang Y. et al., 2021). Possessing a high water content, excellent biocompatibility, and tunable mechanical properties, hydrogels can mimic native tissue microenvironments and respond to physiological stimuli (e.g., pH, temperature, light), enabling spatiotemporally controlled drug release (Cao et al., 2021). Their multifunctionality offers new avenues for enhancing surgical outcomes, minimizing recurrence, and improving drug delivery precision. Recent advances suggest that hydrogel-based systems may serve as platforms for localized therapy, anti-adhesion scaffolding, and post-resection tissue regeneration (Wang H. H. et al., 2024). Thus, the integration of hydrogel technologies into thyroid cancer management represents a novel and promising strategy for addressing existing therapeutic shortcomings and advancing precision medicine.

## 2 Basic review, clinical symptoms, and diagnostic process of thyroid cancer

Thyroid cancer is the tenth most common malignancy globally, with a significantly higher incidence in women, accounting for approximately 75% of cases. The median age at diagnosis is around 50 years, with the highest prevalence observed in individuals aged 16 to 33, particularly among teenagers and young adults. Papillary thyroid cancer (PTC) constitutes about 90% of all thyroid cancer cases, while other histological types include follicular thyroid carcinoma (FTC), Hurthle cell carcinoma (HCC), medullary thyroid carcinoma (MTC), and anaplastic thyroid carcinoma (ATC) (Megwalu and Moon, 2022; Pizzato et al., 2022; Schlumberger and Leboulleux, 2021). The incidence of these subtypes varies geographically and over time. Between 1998 and 2012, the incidence of PTC increased in 25 countries, particularly in several Asian nations, before plateauing. The incidence of FTC remains stable in most countries, with only a slight increase in some regions, while the occurrence of medullary thyroid cancer has remained relatively stable, and undifferentiated thyroid cancer has seen a notable decline (Miranda-Filho et al., 2021). Between 1990 and 2017, the global incidence of thyroid cancer rose by 169%, mortality increased by 87%, and disability-adjusted life years escalated by 75% (Deng et al., 2020). The proliferation of ultrasonography, fine-needle aspiration (FNA) biopsy, and other imaging modalities may have augmented the detection rate of tiny thyroid nodules and early-stage thyroid cancer, resulting in a rise in thyroid cancer diagnoses. While the death rate stayed the same, the incidence rate jumped 15-fold from 1993 to 2011 after thyroid cancer screening was encouraged in South Korea. Studies show that overdiagnosis could explain up to half of the increase in PTC incidence. In the United States, the prevalence of papillary thyroid carcinoma (PTC) across various sizes, stages, and demographic groups has risen, while the death rate for thyroid cancer remains unchanged, suggesting the influence of other factors (Kitahara and Sosa, 2016).

The clinical presentation and diagnostic evaluation of thyroid carcinoma are diverse. Thyroid nodules are commonly detectable by palpation, with approximately 30%–40% of cases identified through this method. A comprehensive assessment of thyroid nodules involves a detailed patient history, physical examination, blood tests, neck ultrasound, and fine-needle aspiration (FNA) biopsy, among other diagnostic procedures. Ultrasound plays a critical role in identifying occult thyroid cancer, with certain characteristics—such as hypoechogenicity, firmness, irregular margins, microcalcifications, an aspect ratio greater than 1, extraglandular invasion, or enlarged cervical lymph nodes—suggesting malignancy. FNA biopsy remains a reliable and frequently employed diagnostic tool. In cases of multinodular goiters, nodules should be biopsied based on their assessed risk of malignancy. Although molecular diagnostic platforms such as ThyroSeq v3 and Afirma have been developed to analyze indeterminate thyroid nodules, their high cost and logistical complexities limit their widespread use, particularly in resource-constrained settings (Kussaibi and Alsafwani, 2023; Liu J. B. et al., 2023).

Moreover, metastases are common in malignant thyroid cancer. Microcalcifications, uneven morphology, multifocality, and capsule invasion in thyroid carcinoma are dependable indicators of lymph node metastases in patients (Li et al., 2020; Mao et al., 2020). Research indicates that lung metastasis frequently occurs in PTC, HCC, poorly differentiated thyroid carcinoma (PDTC), and ATC; bone metastasis is prevalent in follicular thyroid carcinoma (FTC) and MTC; MTC exhibits a high incidence of liver metastasis; multi-organ metastasis is more common in MTC and ATC; and brain metastasis is infrequent (Vuong et al., 2022; Kato et al., 2021; Rafało, 2022; Toraih et al., 2021). Patients with localized lymph node metastasis in the neck exhibit a 5-year survival rate of approximately 70%–90% following surgical excision and postoperative adjuvant therapy, with some achieving prolonged survival; conversely, those with distant metastases to the bones and brain experience a decline in the 5-year survival rate to around 40%–60%, accompanied by a notable decrease in both survival and quality of life.

Genetic (family history, germline mutations), environmental exposures (ionizing radiation, radioactive substances, abnormal iodine levels, environmental pollutants), medical conditions (thyroid nodules, chronic thyroiditis), and lifestyle choices (obesity, iodine consumption, tobacco use, alcohol consumption) define thyroid cancer risk factors. Studies find that early radiation exposure and PTC are strongly correlated. Research confirmed the radiation dose-response link following the Chernobyl accident, showing younger people are more vulnerable and may have consequences spanning more than 30 years (Alsen et al., 2021; Bogović Crnčić et al., 2020; Kitahara and Schneider, 2022). Furthermore, increasing age is associated with a higher risk of morbidity and mortality, with women having a threefold higher incidence than men. Non-toxic nodular goiter and high preoperative blood thyroid-stimulating hormone (TSH) levels have been associated with an increased risk of thyroid carcinoma. Knowing these risk factors helps one to apply strategies for disease prevention.

## 3 Molecular attributes and etiology of thyroid carcinoma

The etiology and molecular characteristics of thyroid cancer vary depending on the histological type. Although PTC is generally associated with an excellent prognosis, there is a notable recurrence rate. PTC is divided into four subtypes based on the molecular level researches: immune-enriched, BRAF-enriched, stromal, and CNV-enriched (Hong et al., 2022). There are many driving mutations in the occurrence and development of PTC, such as RET rearrangements, RAS mutations, and BRAF mutations are examples of common driver mutations (Liu et al., 2024; Pappa et al., 2021; Salvatore et al., 2021; Gilani et al., 2022). A study performed an extensive proteogenomic and metabolomic investigation of 102 Chinese individuals diagnosed with PTC. 97 patients had an average of 74 non-synonymous somatic point mutations and 2 insertions or deletions. BRAF (47%, exclusively V600E mutations) was the predominant mutation, followed by MUC16 (36%), RNF213 (8%), and MSH6 (7%), which also exhibited significant mutation frequencies. It is worth noting that MUC16 and TERT promoter mutations, along with certain gene fusions (e.g., NCOA4-RET), were prevalent in patients exhibiting a high risk of recurrence (Qu et al., 2024). The BRAFV600E initiates the BRAF/MAPK pathway, which promotes PTC by increasing TBX3 expression, promoting the release of CXCR2 ligands, and attracting MDSCs to create an immunosuppressive environment, according to Zhang (Zhang P. et al., 2022). Even though immunosuppression is associated with BRAF mutations, which are more common in classical papillary thyroid carcinoma, intratumoral heterogeneity still affects patient prognosis. In PTC, since RAS gene mutations and thyroid receptor changes are mutually exclusive, it is currently unknown how to predict disease-specific mortality despite the fact that RAS gene mutations are common. TERT mutations increase PTC invasiveness when combined with other driver mutations (Matsuse and Mitsutake, 2023). Mutations in the TERT promoter are associated with poor clinical and prognostic outcomes, and the total mutation load can be used to predict survival (Macerola et al., 2021; Ohori and Nishino, 2023).

In PDTC, the incidence of genetic alterations is influenced by histological criteria and detection tools. The prevalence of BRAF and RAS mutations varies, as do their clinical features. Mutations in the TERT promoter are common in advanced malignancies and associated with an increased risk of metastasis and mortality. EIF1AX mutations are common in PDTC and have a strong correlation with RAS mutations. Their chromosomal copy number abnormalities and gene rearrangements differ from those seen in PTC (Cracolici and Cipriani, 2023; Bandargal et al., 2022). The prevalence of BRAF and RAS mutations in ATC is less than that in differentiated thyroid cancer. Mutations in the TERT promoter and TP53 are prevalent. TP53 mutations are essential for its invasiveness. Numerous other genetic mutations exist. The pathogenesis is associated with genomic instability, and there are four molecular subtypes (Abe and Lam, 2021; Rao and Smallridge, 2023). Research indicates that IGF-1 and IGF-2 released by M2-like tumor-associated macrophages enhance the stemness of anaplastic thyroid carcinoma cells and promote metastasis through the activation of the IR-A/IGF1R-mediated PI3K/AKT/mTOR signaling pathway (Lv et al., 2021). The development of a thyroid cancer model utilizing CRISPR-Cas9 technology revealed that the upregulation of TIMP1, MMP9, and CD44 in genetically modified thyroid progenitor cells facilitates tumor proliferation, while the increased expression of KISS1 and KISS1R in cells harboring BRAF, NRAS, or TP53 mutations correlates with the likelihood of invasion and metastasis. Targeting KISS1R and TIMP1 inhibition may serve as adjunctive therapeutic strategies for undifferentiated thyroid cancer to restoring the RAI uptake capacity of ATC or metastatic lesions (Veschi et al., 2023). MTC is categorized into familial and sporadic forms. The RET proto-oncogene is pivotal. Familial medullary thyroid carcinoma (MTC) frequently exhibits germline RET mutations, while the RET mutation prevalence in sporadic MTC is 44%. Mutations in the RET gene can lead to various clinical manifestations, and the genetic profile of sporadic medullary thyroid carcinoma (MTC) is notably more intricate (Chiacchiarini et al., 2021; Xu et al., 2024). Moreover, PAX8-PPARγ gene rearrangement has been found in a considerable proportion of FTC and a few follicular adenomas (Sakaguchi et al., 2021). Genes associated with the Wnt pathway and the DNA mismatch repair pathway exhibit mutations to differing extents in thyroid cancer (Al-Jundi et al., 2020).

## 4 Conventional management of thyroid carcinoma

The management of thyroid cancer varies according to its stage and histological subtype. For differentiated thyroid carcinoma (DTC), treatment is initiated based on comprehensive risk stratification, which includes physical examination, ultrasonographic assessment, and cytological analysis (Ullmann et al., 2023). In cases of low-risk papillary thyroid carcinoma, active surveillance involving periodic neck ultrasonography has emerged as a potential alternative to immediate intervention. However, this approach remains relatively novel in many countries and is currently undergoing clinical evaluation (Chou et al., 2022; Wei et al., 2022). For patients with low-risk profiles who prefer non-surgical options, minimally invasive image-guided techniques—such as radiofrequency ablation, microwave ablation, and laser ablation—offer promising therapeutic alternatives. Nonetheless, direct comparative studies between these interventional methods and active surveillance strategies are lacking, highlighting the need for further research to establish their relative efficacy and long-term outcomes (Mauri et al., 2021).

Surgical intervention remains the predominant therapeutic approach (Matsuura et al., 2022). The optimal surgical approach for PTC remains a subject of debate, with decisions guided by preoperative factors such as tumor size, multifocality, contralateral nodules, and the risks associated with reoperation. Total thyroidectomy is typically indicated for high-risk cases, whereas thyroid lobectomy is preferred for low-risk tumors. Each approach carries distinct implications for postoperative thyroid function, quality of life, and healthcare costs. Notably, the adoption of thyroid lobectomy has increased in recent years (Nabhan et al., 2021). Research indicates that the health-related quality of life (HRQOL) of patients with DTC at low to moderate risk of recurrence is unaffected by the degree of surgical intervention. Thyroid lobectomy may be preferred over total thyroidectomy (Chen et al., 2022; Schlumberger and Leboulleux, 2021) for better HRQOL in the near term. Although total resection results in a greater incidence of comorbidities, including possible injury to the recurrent laryngeal nerve, the survival and recurrence odds are similar with both.

Medullary thyroid carcinoma (MTC) requires pre-treatment assessment through serum calcitonin determination, carcinoembryonic antigen determination, genetic testing and other diagnostic criteria before the start of treatment. While the extent of lateral lymph node dissection remains debated, total thyroidectomy with bilateral central lymph node dissection is standard in early-stage disease. Conventional chemotherapy offers limited benefit in advanced cases; however, multi-target tyrosine kinase inhibitors such as vandetanib and cabozantinib have demonstrated efficacy in prolonging progression-free survival. Emerging RET-specific inhibitors and ongoing investigations into immunotherapy offer renewed therapeutic promise (Hou et al., 2024; Al-Jundi et al., 2020).

Undifferentiated thyroid carcinoma, the most aggressive subtype, is associated with a poor prognosis and rapid disease progression. Typically presenting as a rapidly enlarging neck mass, it is often unresectable at diagnosis. Median survival is short, with high rates of local recurrence and frequent distant metastases to the lungs, bones, liver, and other organs (Liu et al., 2021). Treatment of anaplastic thyroid carcinoma depends on tumor resectability. Resectable cases are managed with surgery followed by adjuvant radiotherapy and chemotherapy, while unresectable tumors rely primarily on non-surgical modalities. The development of combined BRAF and MEK inhibitor therapy has shown promise in BRAFV600E-mutant tumors. Additionally, targeted agents against the PI3K/AKT/mTOR pathway, immunotherapies, and other novel approaches are under active investigation (Bible et al., 2021).

Consequently, thyroid cancer is predominantly addressed with surgical excision. Nonetheless, since the late 1990s, despite the advent of minimally invasive thyroid surgical techniques such as minimally invasive video-assisted thyroidectomy, robotic-assisted transaxillary thyroidectomy, and transoral endoscopic vestibular approach to the thyroid, the inherent risks associated with thyroid lobectomy and total thyroidectomy remain unchanged (Rossi et al., 2021; Fassas et al., 2021; Scerrino et al., 2022; Tae, 2021). The recurrent laryngeal nerve around the thyroid gland may sustain damage during surgery, resulting in vocal loss, and the many neurovascular systems in the neck, including the internal jugular vein and the accessory nerve, may also be compromised. Trauma to the internal jugular vein results in significant hemorrhage, whereas damage to the accessory nerve leads to impairments in shoulder mobility (Huang et al., 2021). The parathyroid glands are situated next to the thyroid gland. If the surgery involves them, it will impact parathyroid hormone secretion, leading to reduced blood calcium levels and inducing hypocalcemia symptoms such as cramps in the hands and feet, potentially resulting in lasting damage that affects quality of life (Rao et al., 2023). Patients who have undergone total thyroidectomy will experience a loss of thyroid function post-surgery and may require lifelong thyroid hormone replacement therapy. Improper adjustment of alternative doses may lead to symptoms of hypothyroidism or hyperthyroidism, which may have adverse effects on heart health and cause arrhythmia (Mirghani et al., 2024). Moreover, a debate persists on the necessity of total thyroidectomy for low-risk thyroid micro-papillary carcinoma (Hsiao et al., 2022). Neck lymphadenectomy also has dangers. In severe cases, wound infection and prolonged healing may ensue (Baud et al., 2022; Rehell et al., 2022).

RAI therapy can be used to treat residual or recurrent lesions, help with treatment, or ablate remaining tissue (Mayson et al., 2021). However, there are disadvantages to RAI (RAI) therapy, such as decreased salivary production, increased vulnerability to oral infections and dental caries, potential harm to the reproductive system, and injury to the salivary glands that causes xerostomia (De la Vieja and Riesco-Eizaguirre, 2021). Furthermore, following RAI therapy, the capacity of tumor cells in certain DTC patients to absorb iodine-131 diminishes, a condition referred to as RAI refractory thyroid cancer (RAI-RTC) (Liu Y. et al., 2023). This may be related to gene mutations (such as RTK/RAS/BRAF pathways, TERT promoters) and gene rearrangements, resulting in structural and functional abnormalities of the encoded proteins (Chen et al., 2024). A separate study demonstrated that in a prospective randomized phase 3 trial involving patients with low-risk thyroid cancer, the follow-up strategy of abstaining from RAI post-thyroidectomy was shown to be non-inferior to the ablation strategy utilizing RAI (1.1 GBq) regarding the incidence of functional, structural, and biological events over a 3-year period. The quality of life of patients in both groups was comparable (Leboulleux et al., 2022). Targeted medication therapy is associated with higher costs and, in certain individuals, more severe adverse effects, including hypertension, proteinuria, hand-foot syndrome, and diarrhea. These adverse responses not only diminish patients’ quality of life but may also lead to long-term medication resistance (Zhang Y. et al., 2022; Porter and Wong, 2020).

Recent advancements in thyroid cancer therapies have introduced novel treatments, with nanotechnology-assisted approaches offering distinct benefits, particularly the exceptional performance of hydrogels. Since its discovery in the 1960s, the body of research on hydrogels has expanded significantly. Their excellent biocompatibility can avert immune rejection, and they serve as a secure vehicle for nanoparticles delivering anticancer agents (Cui et al., 2021). The improved water absorption and retention help the hydrogel to tightly encapsulate the nanodrugs and precisely control the release rate. Hydrogels’ implantable, injectable, and degradable qualities, along with their stimuli-responsiveness to the tumor microenvironment (such as pH and temperature), facilitate the precise targeted delivery of encapsulated drugs, raise the local concentration of drugs in the tumor. The hydrogel’s degradability guarantees safe carrier metabolism following nanodrug release, hence lowering the potential toxicity of the carrier material itself as well as the systemic toxicity of free drugs. In keeping with the principles of precision medicine, this is highly relevant to the treatment of thyroid cancer (Cao et al., 2021).

## 5 Definition, classification, and design of hydrogels

Hydrogels are three-dimensional polymer networks formed through chemical or physical cross-linking, with the ability to absorb large quantities of water without disintegrating. Based on their origin, hydrogels can be classified into natural polymer hydrogels (e.g., alginate, chitosan, cellulose, pectin, gelatin, hyaluronic acid), synthetic polymer hydrogels (e.g., polyethylene glycol, polyacrylic acid, and their derivatives), and composite hydrogels, which combine the benefits of both natural and synthetic components (Dattilo M et al., 2023; Tan et al., 2021; Nasution et al., 2022).

Each type of hydrogel offers unique advantages in anticancer applications. Alginate-based hydrogels are biocompatible and biodegradable, making them suitable for encapsulating chemotherapeutic agents and immunological adjuvants, thereby enabling controlled drug release and immune modulation (Reig-Vano et al., 2021). Alginate-based 3D *in vitro* models using encapsulated spheroids of C643 and SW1736 anaplastic thyroid carcinoma cells showed higher IC_50_ values for the MEK/Aurora kinase inhibitor BI-847325 compared to 2D monolayers, highlighting their superior predictive value for assessing chemoresistance in anticancer drug testing (Samimi et al., 2021). A self-healing chitosan–PEG hydrogel showed superior thermal shielding compared to saline and non-self-healing hydrogels during thermal ablation of thyroid nodules in a Beagle dog model (Huang et al., 2022), while chitosan-based hydrogels are effective in delivering gene therapy agents, possess antimicrobial properties, and can selectively target tumor cells (Hong et al., 2024). Cellulose-derived hydrogels find diverse applications in extended drug release, tissue engineering, wound healing, biosensing, and food packaging (Aswathy et al., 2022). Hyaluronic acid-based hydrogels are commonly applied in aesthetic procedures, controlled drug release, tissue regeneration, and cell culture (Han S. S. et al., 2022). Nucleic acid-derived hydrogels are utilized for biosensing and gene delivery, while peptide and protein-based hydrogels are employed in tissue engineering and drug delivery. Synthetic polymer hydrogels are extensively used in wound care (Mushtaq et al., 2022), controlled drug delivery systems (Jo et al., 2022), tissue engineering scaffolds, and wastewater treatment. Composite hydrogels integrate the properties of both natural and synthetic materials, finding applications in biomedical fields, food preservation, and the adsorption and remediation of environmental pollutants. These diverse hydrogel types are highlighted in Table 1.

**TABLE 1:** Composition, characteristics, and applications of composite hydrogels.

Classification of components	Subcategory	Characteristics	Application
Polysaccharide-based hydrogels	Alginate-derived hydrogels	Good biocompatibility and biodegradability	Drug sustained release, cell scaffold construction in tissue engineeringetc
	Chitosan-derived hydrogels	Good biocompatibility and antibacterial properties, cationic properties	Wound healing and drug delivery, especially gastrointestinal drug delivery
	Cellulose-based hydrogels	Highly hydrophilic, good biocompatibility, biodegradable, and the properties can be adjusted by adjusting the source of cellulose and the preparation process	In the biomedical field, such as wound dressings and sustained-release drugs; in the environmental field, such as sewage treatment and adsorption of heavy metal ions
	Pectin-based hydrogels	Good biocompatibility and biodegradability, and the unique structure of pectin gives it remarkable gel properties	In the food industry, they are often used as thickeners and preservatives; in biomedicine, they are mostly used as drug delivery carriers and scaffolds for tissue engineering
	Hydrogels based on gelatin	Possess the qualities of minimal immunogenicity, biodegradability, and good biocompatibility. They experience sol-gel transitions at varying temperatures because of their temperature sensitivity	The food sector uses them to enhance the texture and freshness of food, and biomedicine uses them extensively for things like drug-release carriers and wound dressings
	Hyaluronic acid-derived hydrogels	Have excellent moisturizing properties and biocompatibility	They can be used in medical aesthetics for filling, ophthalmic drug delivery, artificial tear preparation, and the treatment of joint diseases
Nucleic acid-based hydrogels		Have the characteristics of sequence programmability, good biocompatibility, and sensitive responsiveness. They can be precisely designed and constructed at the molecular level	They can be used in the field of biosensing for highly specific detection of biomolecules, in drug delivery to achieve intelligent controlled release, and also help to simulate the cell microenvironment in tissue engineering (Zhang Y. et al., 2021)
Peptide- and protein-based hydrogels		Good biocompatibility and biodegradability, high structural and functional diversity, and excellent hydrophilicity	Tissue engineering scaffolds, drug delivery, biosensor fabrication (Davari et al., 2022)
Synthetic polymer hydrogels		With controllable physicochemical properties can precisely load drugs for targeted and sustained release	Precisely deliver drugs, such as encapsulating chemotherapy and gene drugs for targeted sustained release (Wang Z. et al., 2023)
Composite hydrogels		Integrate the advantages of natural and synthetic components	It has outstanding advantages in the field of cancer treatment and is also widely used in tissue repair and other scenarios (Guo et al., 2022)

Hydrogels can be classified according to their responsiveness to external stimuli, including temperature-sensitive, pH-sensitive, light-sensitive, enzyme-sensitive, electric field-sensitive, magnetic field-sensitive, and sound-responsive hydrogels (Ding et al., 2022; Yang and Chun, 2020; Wang and Wang, 2021; Deng et al., 2021; Li Z. et al., 2021), refer to Table 2. This stimulus-responsive nature forms the foundation for numerous applications, particularly in controlled drug delivery systems. Stimuli-responsive *in situ* forming hydrogels (ISFH) represent a category of intelligent materials capable of rapidly forming hydrogels in response to specific stimuli under designated conditions. The mechanism behind ISFH involves changes in the molecular structure or chemical bonds of the hydrogel in response to stimuli such as temperature, pH, light, magnetic fields, mechanical forces, or particular chemicals. These changes cause the polymer or polymer precursor in solution to undergo rapid crosslinking, transitioning into a gel state (Shin et al., 2021). ISFH offer several significant advantages, including remarkable adaptability and the ability to be tailored to meet specific physiological or external conditions. By selecting appropriate stimuli for different scenarios, ISFHs can be customized for precise applications. The *in situ* formation property eliminates the need for complex procedures typically associated with conventional hydrogels, reducing both surgical complexity and tissue trauma. Additionally, ISFHs can be directly administered to tumors using a syringe or catheter, making them a highly versatile tool in medical and therapeutic contexts.

**TABLE 2:** Classification and applications of stimulus-responsive composite hydrogels.

Types of stimuli-responsive properties	Characteristics	Applications
Temperature-sensitive	Swelling or shrinking of the gel state at the critical temperature	Controlled release of drugs in the body, regulation of tissue engineering cells, and temperature monitoring for biosensing; cancer treatment; prevention of postoperative adhesion; targeted drug delivery to the brain through the nose; wound healing; and treatment of osteoarthritis (Fan et al., 2022)
pH-sensitive	Ionic groups have different states depending on the environmental pH, and the degree of swelling changes	In targeted drug delivery in the gastrointestinal tract, biomedical pH detection, and wound dressings
Light-sensitive	Containing light-responsive groups that change their physical or chemical properties in the presence of light	In the fields of light-controlled drug delivery, microfluidic fluid manipulation, light-driven robotics, and imaging-guided therapy, changes in function driven by light
Enzyme-sensitive	Changes in physical and chemical properties due to enzymatic catalysis	Intelligent drug delivery for enzyme-related diseases, enzyme activity detection and regulation of cell-material interactions in enzyme-responsive tissue engineering
Electric field sensitive	Under the action of an electric field, the migration and orientation of internal charged groups lead to changes in shape or size	Artificial muscle construction, MEMS actuation and sensing, and tissue and nerve repair with electrical stimulation
Magnetic field sensitive	Contains magnetic nanoparticles, which change their physical properties under the action of a magnetic field	Magnetic targeting drug delivery, magnetic resonance imaging enhancement, and magnetic control of tissue engineering, where magnetic fields are used to guide positioning and cell regulation
Acoustically sensitive	Physical and chemical properties change in response to sound energy	Ultrasound drug delivery, contrast agent development, and ultrasound-assisted tissue engineering and cell manipulation functions are activated by ultrasound stimulation

The design of hydrogel properties, encompassing chemical composition (e.g., natural or synthetic polymers), cross-linking methods (e.g., covalent, dynamic covalent, or physical cross-linking), viscosity, modifications (e.g., incorporation of cell adhesion peptides), mechanical stiffness, porosity, mesh size, dimensionality (2D or 3D), degradability, and integration with other materials, can influence cell biological behavior, drug delivery effects, metabolism and safety *in vivo* (Cao et al., 2021; Pardeshi et al., 2022; Su et al., 2023). Stable networks formed by chemical bonding control the architecture and properties of hydrogels used for the controlled release of anticancer drugs and cellular carriers (Sapuła et al., 2023). Whereas photo crosslinking induces crosslinking in a hydrogel by light exposure, providing spatial and temporal control, and so enabling the development of sustained-release systems in conjunction with photodynamic therapy, physical cross-linking uses non-covalent interactions to form networks, so facilitating gentle *in situ* and targeted drug delivery. In addition, a proper viscosity helps to control drug release; an unsuitable gelation rate—either too fast or too slow—will affect the therapeutic efficacy; this will affect cell survival and function as well as cancer cell migration. While the rate of degradation affects the regulated degradation and *in vivo* metabolism of drugs, an appropriate pace can ensure controllable release of the drug and guarantee the effectiveness and rational distribution of therapeutic components. An optimal disintegration rate is crucial for cell migration, microenvironment remodeling, therapeutic effectiveness, and biosafety. Integrin-mediated adherence to the hydrogel matrix is necessary for cell migration, survival, and proliferation in hydrogels. Adhesion ligands, including RGD, when included in the hydrogel, improve cell attachment and vitality, thereby affecting cellular behavior (Han Q. et al., 2022). Porosity of the hydrogel affects cell adhesion, proliferation, and drug diffusion, so a balance between these elements is necessary. Changing the structure of the hydrogel (e.g., utilizing electrospinning or microfluidic technologies to create certain configurations) or changing the mechanism of drug inclusion with the hydrogel will help to optimize either (Siboro et al., 2021).

## 6 The function of hydrogels in immunotherapy for thyroid carcinoma

The immune landscape of thyroid cancer comprises both effector and suppressive populations that collectively determine tumour behaviour. In PTC, natural killer (NK) cells accumulate through NKG2D-mediated recognition of MICA/B ligands on tumour cells, whereas in anaplastic thyroid carcinoma (ATC) these cells become functionally exhausted—an impairment that can be reversed by PD-1 blockade (Komatsuda et al., 2023). Dendritic cells similarly infiltrate PTC, process tumour antigens, and prime cytotoxic T-cell responses; their presence diminishes markedly in poorly differentiated and anaplastic lesions, limiting adaptive immunity. The role of B lymphocytes remains equivocal, yet the formation of tertiary lymphoid structures—more common in PTC and influenced by BRAF status—correlates with superior clinical outcomes. Conversely, tumour-associated mast cells and M2-polarised macrophages suppress anti-tumour activity and drive invasion, with M2 macrophage density particularly elevated in ATC. Therapeutic strategies that block macrophage recruitment or promote M1 repolarisation are therefore under active investigation (Shin and Koo, 2022; Zhu et al., 2023; Feng et al., 2023; Tao et al., 2021). Additional immunosuppressive cohorts—including myeloid-derived suppressor cells, neutrophils, and regulatory T and B cells—further attenuate effector responses across differentiated thyroid-carcinoma subtypes, highlighting the need for multifaceted immunomodulatory approaches (Liang et al., 2022).

Thyroid-tumour progression is driven by a constellation of immune-evasion strategies. Neoplastic cells downregulate MHC class I molecules and β2-microglobulin, thereby weakening antigen presentation to cytotoxic T lymphocytes. Concurrent aberrant activation of RET signalling diminishes MHC class II expression, further blunting immune recognition (Ruan et al., 2022). Many tumours also over-express inhibitory checkpoint ligands such as PD-L1—particularly in the BRAFV600E mutational context—curtailing both T-cell and NK-cell activity. The surrounding microenvironment reinforces this immunosuppressive state through high levels of tumour-promoting cytokines (TGF-β, IL-4, IL-10) and reduced concentrations of pro-inflammatory mediators (e.g., IFN-γ, IL-12). Chemokines such as CXCL8 enhance invasion and metastasis, while pro-angiogenic factors support neovascularisation. Finally, enzymes like arginase and IDO1 deplete essential metabolites, creating a tolerogenic niche that enables continued tumour growth and immune escape (Menicali et al., 2020).

Immunotherapeutic strategies in thyroid cancer centre on a defined repertoire of tumour-associated targets. Programmed death-ligand 1 (PD-L1) is highly expressed in aggressive papillary and anaplastic thyroid carcinomas, functioning as a key immune checkpoint whose upregulation enables tumour cells to escape cytotoxic surveillance and accelerate disease progression. Beyond PD-L1, differentiated thyroid carcinomas characteristically display thyroid-specific antigens such as thyroglobulin and the TSH receptor, providing additional immunogenic substrates (Viola et al., 2023). A broader panel of surface markers—including ICAM-1, B7-H3, CEA, and neoantigens generated by BRAFV600E mutation—further expands the targeting landscape. These antigens can be exploited by diverse immunotherapeutic modalities, ranging from checkpoint inhibitors and bispecific antibodies to active vaccination platforms and adoptive cellular therapies, each aiming to amplify tumour-specific immunity while sparing normal tissue (Song et al., 2024; Del Rivero et al., 2020; Pu et al., 2021; Lei et al., 2022).

**FIGURE 1:**
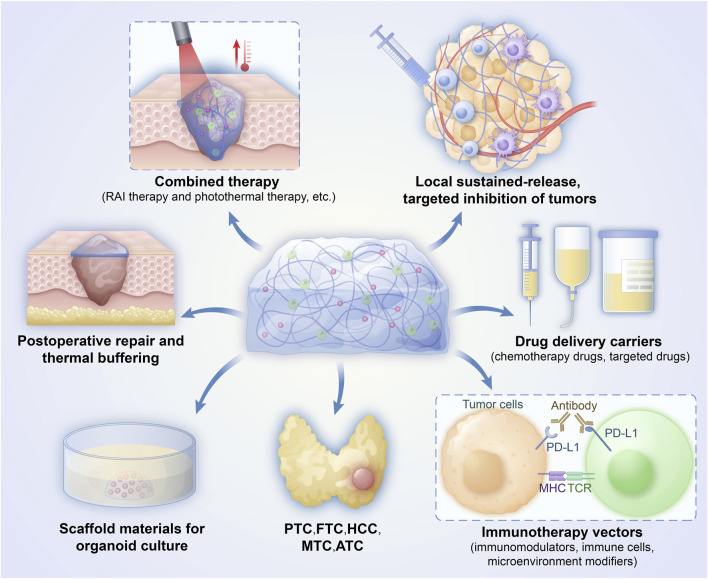
The role of hydrogels in the treatment of thyroid cancer.

Hydrogels provide a multifunctional platform that amplifies each step of the cancer-immunity cycle in thyroid tumours, See Figure 1. Their porous, biocompatible matrix enables direct intratumoral delivery of chemotherapeutics, cytokines, and other immunostimulants, triggering immunogenic cell death and robust antigen release (Mellman et al., 2023). The same architecture attracts and supports dendritic cells, which process these antigens and prime tumour-specific T and B lymphocytes. Sustained release of cytokines and adjuvants from the gel maintains T-cell activation during trafficking and expansion, while the three-dimensional scaffold fosters tertiary lymphoid-structure formation, further enhancing local immune priming. Once infiltrated, effector T cells encounter a hydrogel-conditioned microenvironment that improves tumour-cell recognition and cytolysis, preserving immune-cell viability and maximizing on-target destruction. Collectively, these properties position hydrogels as powerful adjuncts for converting immunologically “cold” thyroid tumours into highly responsive, “hot” lesions (Luo et al., 2021; Santos F et al., 2022).

Chimeric antigen-receptor T-cell (CAR-T) therapy, which bypasses MHC-restricted recognition by equipping T cells with synthetic receptors that directly bind tumour antigens, is now being explored in solid tumours such as thyroid cancer. A recent proof-of-concept study combined CAR-T cells with a degradable hydrogel that simultaneously released the cells and a mitochondrial-autophagy agonist (BC1618). This injectable matrix created a pro-inflammatory niche, enhanced CAR-T expansion, and sustained cytotoxic activity, markedly improving tumour control in a breast-cancer model. Translating this strategy to thyroid malignancies is attractive: the hydrogel’s extracellular-matrix-mimetic structure can guide immune-cell infiltration through dense stromal barriers, while its slow-release profile allows co-delivery of cytokines or chemokines that further promote CAR-T trafficking and persistence within tumour tissue.

Hydrogels can engage in immunotherapy through various effective mechanisms (Correa et al., 2021; Bu et al., 2022). Hydrogels function as versatile immunotherapeutic platforms by engineering a tunable microenvironment that supports the recruitment, proliferation, and activation of key immune populations—including dendritic cells, macrophages, tumour-infiltrating lymphocytes, and cytotoxic T cells. Their injectable matrices enable site-restricted delivery and sustained release of immunoactive payloads, thereby enhancing T-cell persistence and cytolytic potency (Thomas et al., 2021; Grosskopf et al., 2022). Beyond direct cell transport, hydrogels can remodel the tumour milieu: pH-buffering agents, lactate modulators, or L-arginine can be co-encapsulated to counteract metabolic suppression, while STING agonists and cytokines boost innate sensing and adaptive priming. When formulated as vaccine depots, hydrogels co-deliver tumour antigens and adjuvants, fostering local dendritic-cell maturation and robust antigen presentation that drives systemic anti-tumour immunity. Recent modular designs—such as nano-clay/gelatin systems incorporating chemokines, growth factors, and photosensitisers—illustrate how controlled release can simultaneously debulk tumours, expand antigen-presenting cells, and inhibit metastasis. Sustained-release kinetics, intratumoural confinement and the programmed timing design of the hydrogel significantly reduces off-target toxicity (Ji et al., 2023). Looking forward, integration with real-time imaging probes and AI-guided formulation is expected to further personalise hydrogel-based immunotherapies for thyroid cancer and other solid tumours (Li X. et al., 2021; Cui et al., 2021; Zhang X. et al., 2021).

## 7 Hydrogel drug carriers and tailored thyroid cancer medications have the potential to reduce adverse effects and resistance to treatment

Targeted therapy for thyroid cancer provides a powerful new weapon in precision cancer treatment. Its capacity to selectively target certain cancer cell receptors, such as RET and BRAF, explains its capacity to inhibit tumor formation and spread. A multitude of FDA-approved targeted therapies for thyroid cancer is presented in Table 3 (Gild et al., 2021; Puliafito et al., 2022). Although these agents achieve meaningful tumour control, their clinical utility is tempered by adverse-event profiles and the inevitable emergence of resistance. Toxicities commonly involve dermatologic (rash, hand–foot syndrome, alopecia), gastrointestinal (nausea, diarrhoea, constipation, anorexia), cardiovascular (hypertension, QT-interval prolongation), and haematologic (anaemia, leukopenia, thrombocytopenia) systems, in addition to general manifestations such as fatigue and fever and occasional hepatotoxicity or ocular effects (Fallahi et al., 2022; Hamidi et al., 2022; Li D. et al., 2021; Højer Wang et al., 2023). Continuous monitoring and dose modification are therefore essential. Resistance arises through multiple adaptive mechanisms, including upregulation of alternative receptor tyrosine kinases, paradoxical reactivation of the MAPK cascade, PI3K-pathway hyperactivation, and compensatory signalling through JAK/STAT or KEAP1–Nrf2 networks, underscoring the need for next-generation inhibitors and rational combination strategies (Hofmann et al., 2022). Please refer to Table 3.

**TABLE 3:** A summary of FDA-approved targeted therapies for thyroid cancer.

Name	Mechanism of action	Treatment classification
Lenvatinib	Multi-kinase inhibitor that inhibits tumor growth and metastasis by inhibiting various kinase-related signal pathways such as tumor angiogenesis	Treatment of differentiated thyroid cancer refractory to RAI
Sorafenib	Inhibits the kinase activity of kinases involved in tumor cell proliferation and angiogenesis, such as RAF kinase, to prevent tumor cell growth and metastasis	Radioiodine-refractory differentiated thyroid cancer
Vandetanib	Multi-target tyrosine kinase inhibitor that can simultaneously inhibit multiple targets such as RET kinase, VEGFR, and EGFR	Advanced medullary thyroid cancer
Cabozantinib	Can inhibit multiple targets, including RET, MET, and VEGFR-1/2/3, to inhibit the growth of tumor cells, angiogenesis, and metastasis. For differentiated thyroid cancer refractory to RAI, it can also control tumor growth and metastasis by inhibiting the kinases related to tumor angiogenesis and tumor cell proliferation	Advanced medullary thyroid cancer and differentiated thyroid cancer refractory to RAI
Selpercatinib	Acts specifically on these cancer cells carrying RET gene fusion abnormalities, inhibiting their growth, proliferation, and metastasis-related signal pathways, thereby effectively controlling tumor progression	RET gene fusion-positive thyroid cancers, including differentiated thyroid cancer and medullary thyroid cancer
Pralsetinib	A potent and highly selective RET inhibitor that specifically binds to the RET protein and inhibits its kinase activity, thereby blocking the signal pathways related to tumor cell proliferation, survival, and metastasis driven by RET fusions, and achieving the purpose of inhibiting tumor growth	RET fusion-positive thyroid cancers, including differentiated thyroid cancer and medullary thyroid cancer
Dabrafenib	Selectively inhibits the kinase activity of BRAFV600E mutations, effectively blocks the MAPK signaling pathway, and thereby inhibits the growth and spread of tumor cells	BRAFV600E-mutant positive thyroid undifferentiated carcinoma and papillary thyroid carcinoma Dabrafenib must be used in combination with trametinib for the treatment of thyroid cancer
Trametinib	By inhibiting the MEK protein, it can block the downstream MAPK signaling pathway activated by the BRAF mutation, inhibit the proliferation, differentiation, and survival of tumor cells, and slow tumor growth and metastasis	Thyroid undifferentiated carcinoma and papillary thyroid carcinoma with BRAFV600E or V600K mutations

The internal architecture of a hydrogel—its pore size, channel connectivity, and polymer–drug interactions—dictates diffusion kinetics and governs release rates. Hydrophobic, electrostatic, and hydrogen-bonding forces between therapeutic molecules and polymer chains can be finely tuned to restrain burst discharge and achieve prolonged, tumour-confined exposure. This localisation is particularly valuable in thyroid cancer, where systemic chemotherapy or kinase inhibitors often produce dose-limiting toxicities, resulting in failure to attain cytotoxic concentrations at the tumour bed. By moderating peak drug levels and smoothing concentration gradients, hydrogels also lessen cellular stress signalling, thereby delaying the adaptive pathways that underlie acquired resistance.

Recent proof-of-concept systems illustrate the approach. A hyaluronic-acid/carboxymethyl-chitosan matrix loaded with paclitaxel suppressed tumour growth by modulating S100A6 and ARID1A expression (Wang H. H. et al., 2024). Supramolecular peptide nanofibres co-encapsulating dabrafenib and doxorubicin demonstrated high uptake, potent cytotoxicity against BRAFV600E-high cells, and synergistic antitumour activity *in vitro* and *in vivo* (Chen P et al., 2023). Beyond cytotoxics, a PLGA-PEG-PLGA triblock copolymer enabled linear, gastro-resilient release of levothyroxine, reducing dosing frequency and mitigating food-related fluctuations—an important advance for lifelong hormone replacement after thyroidectomy (Movaffagh J et al., 2022). An in situ–forming micelle–hydrogel system (iMHS) further exemplifies this strategy, enabling programmable, sequential release of cisplatin and paclitaxel to achieve improved localized treatment of anaplastic thyroid carcinoma with enhanced intratumoral efficacy, genetic-profile-independent performance, and effective prevention of postoperative recurrence (Yang et al., 2022). *N*α-9-fluorenylmethoxycarbonyl-diphenylalanine (Fmoc-FF) peptide-based nanogels also highlight this potential, enabling the co-delivery of hydrophilic (doxorubicin) and hydrophobic (curcumin) agents to thyroid cancer cells with stable, sustained release, efficient intracellular delivery, and the potential to reduce toxicity while enhancing therapeutic efficacy (Gallo et al., 2025). Collectively, these platforms underscore the capacity of hydrogels to enhance therapeutic index, sustain drug pressure within the tumour, and curb the emergence of resistance.

## 8 Hydrogel bioink promotes thyroid cancer functional regeneration and immune regulation

Hydrogel-based bioinks are pivotal to 3-D bioprinting and organoid engineering because they replicate the biochemical and mechanical cues of the native extracellular matrix (ECM) while maintaining high cell viability. In oncology, these materials enable construction of faithful three-dimensional tumour surrogates—ranging from osteosarcoma to customised thyroid-cancer matrices—that capture cell-matrix and cell-cell dynamics unattainable in monolayer culture. Tissue-engineering efforts now leverage hydrogels to restore thyroid function lost after extensive thyroidectomy. Blending natural or synthetic hydrogels with primary thyrocytes or stem-cell-derived progenitors produces a microenvironment that mirrors the gland’s elasticity, architecture, and growth-factor milieu. Decellularised thyroid ECM (TEM) hydrogels, for example, preserve native proteins and endogenous signals; within these scaffolds, thyroid epithelial cells self-organise into follicular structures and secrete physiological levels of thyroid hormones (Ruchika et al., 2024). Hydrogels can also be printed into immunomodulatory constructs. Sodium-alginate/gelatin bioinks have been engineered to enhance NK cell proliferation and activity directly at the tumour site. When loaded with EGFR-specific CAR-NK cells, these scaffolds markedly improve cytotoxicity, illustrating how bioprinting can overcome trafficking barriers that limit conventional cell therapies. Finally, transplantable thyroid organoids derived from human embryonic stem cells and stabilised within supportive hydrogels have restored euthyroid status in radioiodine-ablated, hypothyroid mouse models. Collectively, hydrogel-based 3-D printing and organoid technologies are advancing from sophisticated disease models to regenerative and immunotherapeutic solutions for thyroid cancer (Ogundipe et al., 2022; Zhang et al., 2024; Kim et al., 2023; Romitti et al., 2022).

## 9 Thyroid cancer treatment with hydrogels and photothermal therapy

When used in conjunction with other treatments, such as photothermal therapy, hydrogels can provide even more effective results. While photothermal treatment (PTT) can kill tumor cells, it can also harm healthy tissue in the area. Hydrogels have several applications in PTT, including local temperature maintenance, thermal damage reduction, and the ability to enable numerous treatments. Numerous novel photothermal agents that have been investigated in conjunction with hydrogels have demonstrated promising anti-tumor outcomes (Yue et al., 2022). Hydrogel-based platforms serve as an effective foundation for photodynamic therapy (PDT), which employs photosensitizers to generate reactive oxygen species and kill tumor cells. However, the photosensitizers themselves have certain disadvantages. By increasing the biocompatibility and local concentration of photosensitizers, hydrogels can enhance the efficacy of PDT (Gan et al., 2023). Zhao developed a hydrogel composed of low-toxicity RAI-131-sodium alginate-indocyanine green (^131^I-ALG-ICG) for the combination of radionuclide and photothermal therapy of thyroid cancer. It serves effectively for the dual application of photothermal therapy and radionuclide therapy. Additionally, it has a strong ability to fixate ICG, which significantly increases the concentration of photothermal agents in the tumor while lowering the possibility of negative effects from ICG diffusion to surrounding tissues (Zhao, 2021). Hao developed a 3D-printed Cu/Ag scaffold for local cancer treatment by studying the therapeutic effect of an Ag-based scaffold crosslinked with Cu^2+^ on thyroid cancer cells and organoids under NIR irradiation. In the study, six organoid models of thyroid cancer were effectively created. Cu/AG + NIR treatment resulted in significant cell death in the organoids, as demonstrated by calcein-AM/PI staining, suggesting that the Cu/AG scaffold had a chemophotothermal impact (Wei et al., 2023).

## 10 Hydrogel's function in postoperative care and thyroid surgery

Minimally invasive as treatment is, image-guided thermal ablation of the thyroid can nonetheless cause heat injury to delicate tissues in the area. The 5% glucose-assisted water separation method that is now used in clinical settings is either helpful or harmful. Zheng et al. developed a multifunctional hydrogel (HA-Dc) composed of hyaluronic acid that is employed for water separation (Zheng et al., 2024). In addition to better tissue retention, stability, and thermal protection, it was shown to have outstanding injectability. This hydrogel technique can regulate heat transmission and serve as a thermal buffer while thermally ablating thyroid cancer, avoiding the overheating of adjacent healthy tissues.

Post-thyroidectomy, adhesion of cervical tissues is prevalent, impairing swallowing and vocal function and complicating subsequent surgical interventions. Hydrogel sheets or gel formulations derived from natural polysaccharides, such as sodium alginate and cellulose, are utilized on surgical wounds post-operation. They inhibit fibrin exudation and excessive fibroblast proliferation and adhesion between wounds through the physical barrier effect. Clinical investigations indicate that the occurrence of neck adhesions in patients receiving sodium alginate hydrogel for anti-adhesion was approximately 50% lower than in the control group 3 months post-surgery. Swallowing dysfunction markedly improved, enhancing patients’ quality of life post-surgery (Wang Y. et al., 2023).

Postoperative application of ice compresses and nanosilver hydrogel care led to reduced skin temperatures, diminished hematoma incidence, a progressive decline in SF-MPQ pain scores, and enhanced comfort in patients undergoing thyroid surgery, hence alleviating their psychophysiological stress (Shi and Wang, 2023). An inventive type of wound dressing is nanosilver antibacterial hydrogel. In patients recovering from injuries, nanosilver ions can promote epithelialization and dramatically reduce infection. High water content, superior permeability, hydrophilicity, self-regulating wound wetness, and promoting the growth of new epithelium are all characteristics of hydrogels. Postoperative wound recovery can be effectively achieved using nanosilver antibacterial hydrogel dressings (Dong et al., 2022). A manganese-loaded, pH-responsive DNA hydrogel incorporating a thyroglobulin-specific aptamer further exemplifies these advanced strategies, enabling targeted magnetic resonance imaging of thyroid tumors with extended tumor retention and enhanced imaging contrast for improved diagnostic precision (Hu et al., 2025).

## 11 The application of hydrogels in RAI treatment of thyroid cancer

For a long time, RAI has been used to treat thyroid cancer, and it is essential for improving the overall survival rate of patients with metastatic thyroid cancer. The sodium/iodine symporter (NIS) is a key player in the treatment of thyroid cancer with RAI (^131^I^−^), according to research. Through the selective uptake of radioisotopes by NIS, ^131^I^−^ treatment can successfully target metastases and residual malignant cells following thyroid cancer surgery (Ravera et al., 2022). However, there are still issues with precision tumor therapy, including as insensitivity in some cancers, limited retention time at the tumor site, ionizing harm to non-targeted organs, insufficient targeting of other cancers, and potential side effects. Hydrogels can provide a stable medium for the storage and release of RAI, increase its concentration at the tumor site, enhance therapeutic efficacy, and lessen damage to nearby normal tissues because of their strong hydrophilic characteristics and extended retention in tumor tissue.

Radiotherapy and chemotherapy can create thermosensitive micelle-hydrogel complexes that sequester RAI and chemotherapeutic agents within tumor tissue to impede tumor proliferation. Many patients integrate radiation with immunotherapy. A specialized immunotherapy platform utilizing particular complexes and radiation can regulate tumor proliferation and metastasis. Integrated systems including many components have demonstrated anti-tumor effects and immune memory responses across distinct tumor types. Hydrogels enhance the precision of RAI in targeting cancers, hence minimizing radiation exposure to healthy tissues and augmenting the safety of the therapy. The combined use of several therapeutic modalities and RAI emission may enhance tumor cell lethality, hence boosting therapeutic efficacy and providing a more promising cancer management approach (Guo et al., 2023).

## 12 Summary and prognosis-hydrogel-focused perspective

Thyroid cancer incidence continues to rise, yet established options—surgery, radioactive-iodine ablation, endocrine manipulation, and multi-kinase inhibition—remain hampered by peri-operative risks, systemic toxicity, and acquired drug resistance. Hydrogels have now moved from ancillary biomaterials to central therapeutic platforms that can address these specific limitations. Their highly hydrated, tunable 3-D networks allow (i) site-confined, sustained delivery of chemotherapeutics, TKIs, or ^131^I; (ii) dynamic modulation of the tumour–immune microenvironment, having the potential to promote the transformation of “cold” tumors into a “hot” state niches; and (iii) matrix-mimetic support for post-resection tissue repair, simultaneously preventing cervical adhesions.

Because the thyroid lies in a densely innervated, vascular neck compartment, hydrogels designed for this indication must satisfy three stringent design criteria: rapid *in situ* gelation with precise anatomical conformity, ultra-low immunogenicity, and on-demand payload release triggered by local pH, enzymatic activity, or mild photothermal input. Emerging stimulus-responsive and composite hydrogels already have made remarkable progress and have demonstrated enhanced tumour retention, reduced off-target exposure, and synergism with photothermal or RAI regimens in pre-clinical models.

Key obstacles remain. Optimising degradation kinetics without sacrificing mechanical integrity, maximising drug-loading efficiency for hydrophobic TKIs, and mapping hydrogel–immune–tumour crosstalk *in vivo* are immediate research priorities. Clinical translation is further limited by a paucity of multi-centre trials to validate long-term safety and comparative efficacy.

Future directions include smart multifunctional systems that co-encapsulate dual-target inhibitors (BRAFV600E + MEK), checkpoint-blocking antibodies or PROTACs, and imaging probes for real-time theranostics. Integration with oncolytic-virus platforms and single-cell-omics-guided patient stratification could unlock personalised hydrogel therapies, particularly for aggressive ATC and refractory metastatic disease.

In conclusion, hydrogels offer a modular, biocompatible, and multifunctional strategy uniquely suited to the anatomical and biological challenges of thyroid cancer. With continued material innovation and rigorous clinical evaluation, hydrogel-based therapeutics hold the potential to redefine locoregional control, mitigate systemic toxicity, and ultimately improve survival and quality of life for patients with thyroid malignancies.

## Glossary

AKT: Protein Kinase B
ALG-ICG: Alginate-indocyanine green
ATC: Anaplastic carcinoma
BRAF: B-Raf Proto-Oncogene
CAR: Chimeric antigen receptor
CNV: Copy Number Variation
CRISPR-Cas9: Clustered Regularly Interspaced Short Palindromic Repeats-CRISPR Associated Protein 9
CXCR: C-X-C Chemokine Receptor
ECM: Extracellular matrix
EGFR: Epidermal Growth Factor Receptor
FDA: Food and Drug Administration
FNA: Fine-needle aspiration
FTC: Follicular thyroid carcinoma
HCC: Hurthle cell carcinoma
HRQOL: Health-related quality of life
IGF: Insulin-Like Growth Factor
ISFH: In situ forming hydrogels
JAK: Janus Kinase
KEAP: Kelch-like ECH-associated Protein
KISS: Kisspeptin
MAPK: Mitogen-Activated Protein Kinase
MDSC: Myeloid-Derived Suppressor Cells
MSH: MutS Homolog
MTC: Medullary thyroid carcinoma
MUC: Mucin
NCOA: Nuclear Receptor Coactivator
NIS: Sodium/iodide Symporter
NK: Natural Killer cell
NRAS: Neuroblastoma RAS Oncogene
PAX: Paired Box
PD-1: Programmed death-1
PDT: Photodynamic therapy
PDTC: Poorly differentiated thyroid carcinoma
PI3K: Phosphatidylinositol 3 - Kinase
PPAR: Peroxisome Proliferator-Activated Receptor
PROTAC: Proteolysis-targeting chimera
PTC: Papillary thyroid cancer
PTT: Photothermal treatment
QT: QT interval
RAI: Radioactive iodine
RAS: Rat Sarcoma
RET: Rearranged during Transfection
RNF: Ring Finger Protein
RTC: Refractory thyroid cancer
RTK: Receptor Tyrosine Kinase
STAT: Signal Transducer and Activator of Transcription
STING: Stimulator of interferon genes
TBX: T-Box Transcription Factor
TEM: Thyroid extracellular matrix
TERT: Telomerase Reverse Transcriptase
TIMP: Tissue Inhibitor of Metalloproteinases
TKI: Tyrosine kinase inhibitor
TOR: Target Of Rapamycin
TP: Tumor Protein
TSH: Thyroid-stimulating hormone.
